# Integration of Single-Cell RNA Sequencing and Bulk RNA Sequencing Data to Establish and Validate a Prognostic Model for Patients With Lung Adenocarcinoma

**DOI:** 10.3389/fgene.2022.833797

**Published:** 2022-01-27

**Authors:** Aimin Jiang, Jingjing Wang, Na Liu, Xiaoqiang Zheng, Yimeng Li, Yuyan Ma, Haoran Zheng, Xue Chen, Chaoxin Fan, Rui Zhang, Xiao Fu, Yu Yao

**Affiliations:** Department of Medical Oncology, The First Affiliated Hospital of Xi’an Jiaotong University, Xi’an, China

**Keywords:** ScRNA-seq, prognosis, prognostic model, NMF, lung adenocarcinoma

## Abstract

**Background:** Lung adenocarcinoma (LUAD) remains a lethal disease worldwide, with numerous studies exploring its potential prognostic markers using traditional RNA sequencing (RNA-*seq*) data. However, it cannot detect the exact cellular and molecular changes in tumor cells. This study aimed to construct a prognostic model for LUAD using single-cell RNA-*seq* (scRNA-*seq*) and traditional RNA-*seq* data.

**Methods:** Bulk RNA-*seq* data were downloaded from The Cancer Genome Atlas (TCGA) database. LUAD scRNA-*seq* data were acquired from Gene Expression Omnibus (GEO) database. The uniform manifold approximation and projection (UMAP) was used for dimensionality reduction and cluster identification. Weighted Gene Correlation Network Analysis (WGCNA) was utilized to identify key modules and differentially expressed genes (DEGs). The non-negative Matrix Factorization (NMF) algorithm was used to identify different subtypes based on DEGs. The Cox regression analysis was used to develop the prognostic model. The characteristics of mutation landscape, immune status, and immune checkpoint inhibitors (ICIs) related genes between different risk groups were also investigated.

**Results:** scRNA-*seq* data of four samples were integrated to identify 13 clusters and 9cell types. After applying differential analysis, NK cells, bladder epithelial cells, and bronchial epithelial cells were identified as significant cell types. Overall, 329 DEGs were selected for prognostic model construction through differential analysis and WGCNA. Besides, NMF identified two clusters based on DEGs in the TCGA cohort, with distinct prognosis and immune characteristics being observed. We developed a prognostic model based on the expression levels of six DEGs. A higher risk score was significantly correlated with poor survival outcomes but was associated with a more frequent *TP53* mutation rate, higher tumor mutation burden (TMB), and up-regulation of *PD-L1*. Two independent external validation cohorts were also adopted to verify our results, with consistent results observed in them.

**Conclusion:** This study constructed and validated a prognostic model for LUAD by integrating 10× scRNA-*seq* and bulk RNA-*seq* data. Besides, we observed two distinct subtypes in this population, with different prognosis and immune characteristics.

## Introduction

Lung cancer is one of the most common incident cancers and the leading cause of cancer-related death worldwide (Chen et al., 2016). As the most predominant pathological subtype, lung adenocarcinoma (LUAD) makes up more than 40% of lung cancer cases (Travis et al., 2015; Neal et al., 2019). Although promizing progress has been made in the screening, diagnosis, and management of LUAD patients in recent decades, it remains a lethal disease because a significant fraction of patients is diagnosed at the advanced disease stage (Denisenko et al., 2018; Lurienne et al., 2020). It is reported that more than 60% of newly diagnosed patients present locoregional or distant metastases at the time of detection (Brozos-Vázquez et al., 2021), with overall survival (OS) less than 5 years (Denisenko et al., 2018). With the rapid development of cancer genomics in recent decades, more and more gene alteration has been identified as an effective treatment target for LUAD. The majority of LUAD patients with driver gene mutation can benefit from molecular targeted therapy, such as epidermal growth factor receptor (*EGFR*)- tyrosine kinase inhibitors (TKIs), anaplastic lymphoma kinase (*ALK*)-TKIs (Yi et al., 2021a), and recently *KRAS* (Uras et al., 2020) and c-*MET* (Zhang et al., 2018) inhibitors. However, there is still part of patients who cannot get rid of the fate of resistance to these drugs due to secondary mutation in tumors. Recently, immune checkpoint inhibitors (ICIs) that target cytotoxic T lymphocyte-associated protein 4 (*CTLA4*), programmed death 1 (*PD1*), and programmed death-ligand 1 (*PD-L1*) have shown promising effects in various malignancies, including LUAD (Chen Y. et al., 2021; Huang et al., 2021). Unfortunately, not all patients can benefit from ICIs intervention, with a lower overall response rate observed in clinical practice. Therefore, there is an urgent need to identify potential prognostic and predictive biomarkers that could precisely stratify patients and recognize patients who will respond to treatment.

In recent decades, a growing body of studies explored potential prognostic markers of LUAD using traditional RNA sequencing (RNA-*seq*) data and have improved our understanding of tumor occurrence and development (Chen et al., 2020). For instance, Yi et al. developed a prognostic model to predict LUAD patients’ survival and response to immunotherapy based on 17 immune-related genes (Yi et al.). Liang et al. also constructed a prognostic model for these patients based on seven ferroptosis-related genes (Liang et al.). Besides, our previous study also identified an autophagy-related long non-coding RNA signature as a prognostic biomarker for LUAD patients (Jiang et al., 2021). Despite the promising predictive power has been observed in the above studies, these prognostic signatures are based on traditional RNA-*seq*, which cannot detect the exact cellular and molecular changes in tumor cells because it mainly concentrates on the “average” expression of all cells in a sample (Chen et al., 2020).

Recently, single-cell RNA-*seq* (scRNA-*seq*) has been used to investigate the transcriptome of different cell types as an innovative technology (Chen et al., 2020). It uses optimized next-generation sequencing technologies to define the global gene expression profiles of single cells, thus facilitating dissection of the previously hidden heterogeneity in cell populations (Liang et al., 2021). Given this advantage, numerous studies have focused on identifying novel biomarkers for malignancies by integrating scRNA-*seq* and traditional RNA-*seq* (Zhang et al., 2019; Chen et al., 2020; Liang et al., 2021). This study aimed to construct a prognostic model for patients with LUAD by integrating scRNA-*seq* and traditional RNA-*seq* data, with two external validation cohorts being adopted to verify its risk stratification ability. Besides, we also identified two different population subtypes using non-negative matrix factorization (NMF), with distinct prognosis and immune characteristics observed. We believe our findings will provide potential prognostic biomarkers and therapeutic targets for LUAD.

## Materials and Methods

### Raw Data Acquisition

10× scRNA-*seq* data of two LUAD samples (T1 and T2) and two normal samples (N1 and N2) were downloaded from the GSE149655 series, which included 2,642 cells, 3,203 cells, 4,243 cells, and 2,466 cells for each sample. LUAD bulk RNA-*seq* data, mutation data, and clinicopathological characteristics were downloaded from the TCGA database. Besides, we also downloaded progression-free survival (PFS) records of these patients from UCSC Xena (https://xena.ucsc.edu/). The human. gtf file was adopted to raw matrix annotation. Furthermore, GSE31210 and GSE13213 cohorts were also acquired from the Gene Expression Omnibus (GEO) (https://www.ncbi.nlm.nih.gov/) database to serve as independent external cohorts for risk model validation. The detailed clinical characteristics of patients in the TCGA and GEO cohorts are summarized in Supplementary Table S1.

### scRNA-*Seq* Data Processing and Analysis

The 10× scRNA-*seq* data were processed according to the following steps: 1) R software, “Seurat” package (Macosko et al., 2015) was adopted to convert 10× scRNA-*seq* data as a Seurat object; 2) quality control (QC) of the raw counts by calculating the percentage of mitochondrial or ribosomal genes and excluding low-quality cells; 3) the “FindVariableFeatures” function was adopted to filter the top 2000 highly variable genes after QC; 4) principal component analysis (PCA) was performed based on the 2000 genes, and uniform manifold approximation and projection (UMAP) (Becht et al., 2018) was used for dimensionality reduction and cluster identification; 5) the “Find All Markers” function was exploited to identify significant marker genes for different clusters by setting log_2_ [Foldchange (FC)] as 0.3 and min.pct as 0.25; and 6) R software, “SingleR” package (Aran et al., 2019) was applied to cluster annotation to recognize different cell types. Next, we performed Fisher’s exact test to identify potential significant cell types between tumor and normal samples. We calculated the FC value of each cell type in tumor and normal samples and determined the cell types with FC> 4 or FC <0.25, *p*-value < 0.05 as the key cell types. Furthermore, we performed functional enrichment analysis for the identified hub cell types using R software, “ReactomeGSA” package (Griss et al., 2020). We used the “analyze_sc_clusters” function for enrichment analysis and extracted the results through the “pathways” function. R software, “monocle” package (Borcherding et al., 2019) was adopted to cell trajectory and pseudo-time analysis, with the method “DDRTree” being used for dimensionality reduction. Subsequently, the statistical method “BEAM” was used to calculate the contribution of genes during cell development, and the top 100 genes were selected for visualization. Ultimately, R software, “CellChat” (Jin S. et al., 2021) and “patchwork” packages were adopted for cell-cell communication analysis and network visualization.

### Differentially Expressed Genes Identification and Functional Enrichment Analysis

Differential expression analysis was performed to filter differentially expressed genes (DEGs) in the TCGA cohort by using the R software, “limma” package, with |log_2_FC| >1.0 and false discovery rate (FDR) < 0.05 being used as cut-off value. The volcano plot was generated to visualize the distribution of the identified DEGs. Subsequently, Kyoto Encyclopedia of Genes and Genomes (KEGG) and Gene Ontology (GO) analyses were exploited to investigate the most significantly enriched pathways and biological processes of the DEGs using R software, “clusterProfiler” package.

### Weighted Gene Correlation Network Analysis

Weighted Gene Correlation Network Analysis (WGCNA) was utilized to filter hub genes in DEGs via R software, “WGCNA” package. WGCNA is divided into expression cluster and phenotypic correlation analyses (Langfelder and Horvath, 2008). It mainly includes four steps: calculation of correlation coefficient between genes, determination of gene modules, co-expression network, and correlation between modules and traits (Langfelder and Horvath, 2008). In the process of co-expression network construction, soft thresholding power β was selected as the lowest power with which fit index of scale-free topology reached 0.90. The modules were presented together via dendrogram after the process of clustering. Subsequently, the module-trait heatmap was generated to further identify the most significant DEGs in LUAD development by comparing their correlation coefficients and *p* values. Ultimately, we selected the intersection genes among the marker genes and DEGs found in WGCNA for further analysis.

### Sample Clustering Using Non-Negative Matrix Factorization Algorithm

Non-negative matrix factorization (NMF) was carried to divide patients into different subtypes according to the following steps: 1) the univariate Cox regression analysis was performed to identify potential prognostic DEGs *via* R software, “survival” package; 2) sample clustering through “brunet” method in R software, “NMF” package; 3) according to parameters such as cophenetic, dispersion, and silhouette, the optimal number of the cluster was identified to classify patients into different subtypes; and 4) the consensus heatmap was generated in accordance with the above optimal cluster number to view the distribution characteristic among different subtypes. Then, we also explored the relationship between different clusters and OS and PFS. Besides, the MCPcounter algorithm was adopted to estimate the infiltration of the immune cells between different clusters. We also investigated the association between clusters and six immune subtypes identified in a previously published study (Tamborero et al., 2018).

### Prognostic Model Construction and Validation

First, the univariate Cox regression analysis was performed to identify potential prognostic DEGs. Variables with a *p*-value < 0.01 were selected into the Least Absolute Shrinkage and Selection Operator (LASSO) regression analysis to reduce the number of genes in the final risk model through R software, “glmnet” package. Ultimately, genes in the LASSO regression were selected into the multivariate Cox regression analysis and therefore constructed the prognostic model according to the following formula:

$$\text{risk}\;\text{score}=\sum_{i=1}^{\text{k}}\beta i*\mathrm{expi}$$ (1)

In the formula, “βi” represents the coefficient of the selected genes in the multivariate Cox analysis and “expi” refers to its expression value. All patients were divided into high- and low-risk groups according to the median value of risk score. Survival curves and risk plots were generated to visualize the survival difference and status for each patient via R software, “survminer” and “ggrisk” packages. Besides, we used R software, “timeROC” package to draw the receiver operating characteristic (ROC) curves to evaluate the performance of risk score in predicting 1-, 3-, and 5 years OS of LUAD patients. Additionally, GSE31210 and GSE13213 cohorts were used as independent external cohorts to validate the utility of the prognostic model.

### Clinical Relevance, Mutation Landscape, and Enrichment Analysis Between High- and Low-Risk Groups

Next, we investigated the association between the risk score and clinicopathological characteristics of patients in the TCGA cohort. Furthermore, we adopted Cox regression analysis to determine whether the risk score could be an independent prognostic factor for LUAD patients via R software, “survcomp” package. At the same time, R software, “forestplot” package was used to draw forest plots of the univariate and multivariate Cox regression analyses. Gene set enrichment analysis (GSEA) was then performed to identify the most significantly enriched pathways between high- and low-risk groups through R software, “org.Hs.eg.db,” “clusterProfiler,” and “enrichplot” packages. In addition, two waterfall plots were generated to explore the detailed gene mutation characteristics between high- and low-risk groups *via* “oncoplot” function in R software, “maftools” package.

### Immune Cells Infiltration and Immune Function Status Between High- and Low-Risk Groups

Then, single-sample gene set enrichment analysis (ssGSEA) (Rooney et al., 2015) was adopted to estimate the infiltrating score of immune cells and the activity of immune-related pathways using R software, “GSVA” and “GSEABase” packages. The Wilcoxon rank-sum test was used to compare the statistical difference between high- and low-risk groups. Besides, we also investigated the correlation between risk score and immune checkpoint inhibitors (ICIs) related genes expression levels and tumor mutation burden (TMB), with R software, “ggplot2” package being adopted for visualization.

### Statistical Analysis

The non-parameter Wilcoxon rank-sum test was used to examine the relationship of continuous variables between the two groups. The LASSO regression and Cox regression analyses were used for predictive model development. Kaplan-Meier survival analysis was used to test the survival difference between different risk groups. A log-rank test was adopted to examine the statistical difference. A two-sided *p*-value < 0.05 was considered significant. All analyses were conducted in R software (version 4.1.1) for windows 64.0.

## Results

### scRNA-*Seq* and Cell Typing of Normal and Lung Adenocarcinoma Lung Samples

**FIGURE 1:**
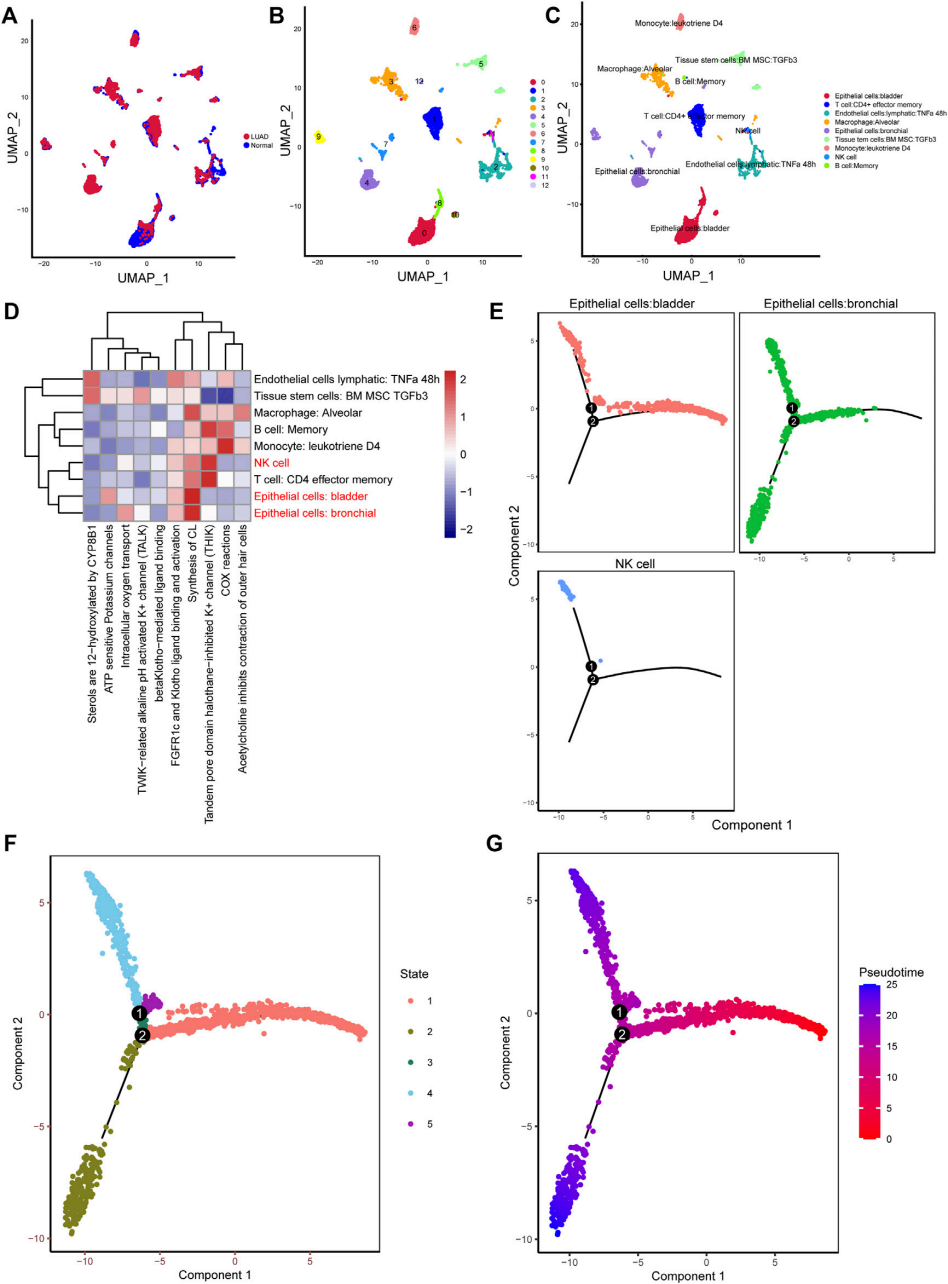
Different clusters annotation and cell types identification in LUAD 10× scRNA-*seq* data. **(A–C)** Clusters annotation and cell types identification via UMAP; **(D)** Functional enrichment analysis for the identified hub cell types using “ReactomeGSA” package; **(E–G)** Cell trajectory and pseudo-time analysis for the identified hub cell types. LUAD, lung adenocarcinoma; scRNA-*seq*, single-cell RNA sequencing; UMAP, uniform manifold approximation and projection.

**FIGURE 2:**
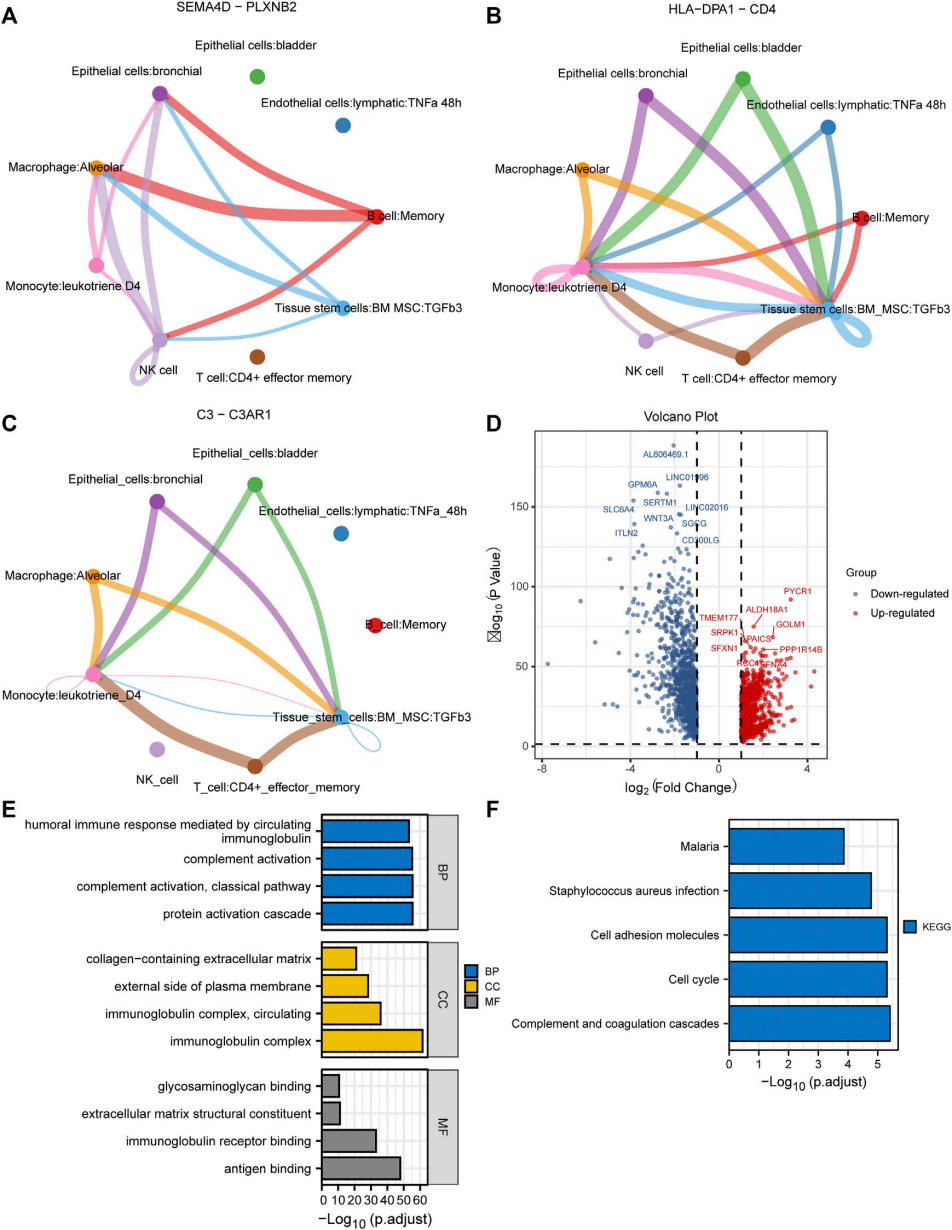
Cell-cell communication network and identification of DEGs in TCGA cohort. **(A–C)** Cell-cell communication network identified that SEMA4D—PLXNB2, HLA−DPA1—CD4, and C3—C3AR1 play crucial roles in the communication network; **(D)** The volcano plot to show the up-regulated and down-regulated DEGs in TCGA cohort; **(E,F)** GO and KEGG enrichment analysis of the identified DEGs. DEGs, differentially expressed genes; TCGA, The Cancer Genome Atlas; GO, Gene Ontology; KEGG, Kyoto Encyclopedia of Genes and Genomes.

10× scRNA-*seq* data of two LUAD and two normal samples were downloaded from the GSE149655 dataset. A total of 8,170 cells were identified after QC, as shown in Supplementary Figure S1A. We visualized the top 20 highly variable genes in Supplementary Figure S1B. Thirteen distinct clusters were identified after PCA and UMAP analysis (Figures 1A,B). Then “SingleR” package was adopted to cluster annotation, with UMAP being used to visualize the cell types after dimensionality reduction. Overall, we identified nine cell types in this step, including bladder epithelial cells, CD4^+^ effector memory T cell, lymphatic endothelial cells, alveolar macrophage, bronchial epithelial cells, tissue stem cells, monocyte, NK cells, and memory B cell (Figure 1C). Of these, NK cells, bladder epithelial cells, and bronchial epithelial cells were identified as significant cell types. ReactomeGSA functional enrichment analysis suggested that these cell types mainly are involved in intracellular oxygen transport, FGFR1c and Klotho ligand binding and activation, and synthesis of cardiolipin (CL) (Figure 1D). Then, “monocle” package was exploited to analyze the cell trajectory and pseudo-time of the identified three significant cell types. We observed that NK cell only corresponds to state 4, while bronchial epithelial cells occurred in the whole state (Figures 1E–G). We then calculated the contribution of genes during cell development, and the top 100 genes were selected for visualization (Supplementary Figure S2A). We investigated the cell-cell communication network by calculating communication probability (Supplementary Figure S2B). Furthermore, we inferred the cell-cell communication network based on specific pathways and ligand-receptors. We identified that SEMA4D—PLXNB2 (Figure 2A), HLA−DPA1—CD4 (Figure 2B), and C3—C3AR1 (Figure 2C) play crucial roles in the communication network.

### Identification of Differentially Expressed Genes in Bulk RNA-*Seq* Data

**FIGURE 3:**
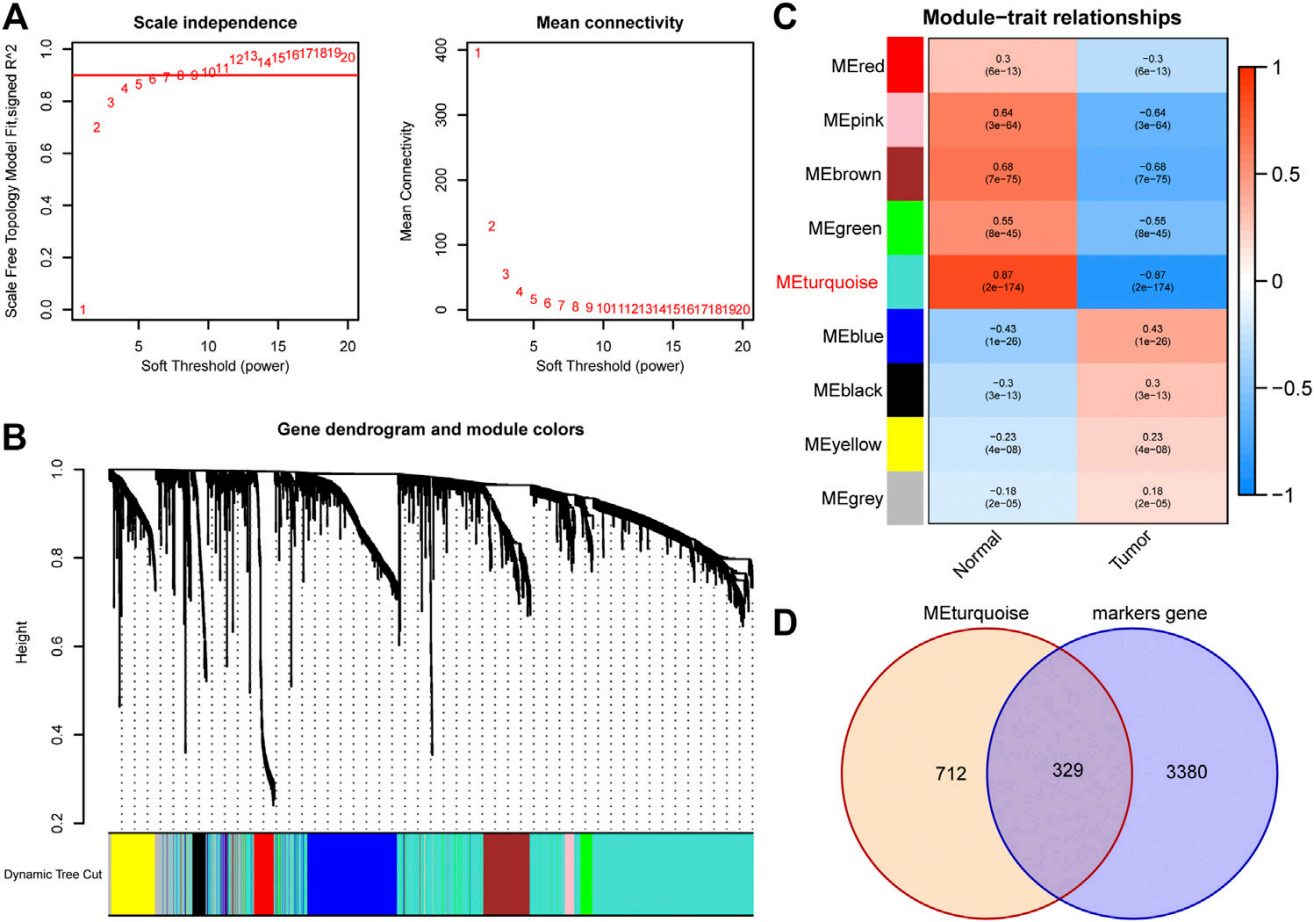
Identification of hub DEGs that participate in LUAD development through WGCNA. **(A)** The scale-free fit index for soft thresholding powers. The soft thresholding power β in the WGCNA was determined based on a scale-free *R* ^2^ (*R* ^2^ = 0.90). The left panel illustrates the relationship between β and *R* ^2^. The right panel illustrates the relationship between β and mean connectivity. **(B)** A dendrogram of the DEGs clustered based on different metrics. **(C)** A heatmap illustrates the correlation between different gene modules and clinical traits (normal vs. tumor); **(D)** The Venn plot to identify common DEGs between WGCNA module genes and marker genes. DEGs, differentially expressed genes; LUAD, lung adenocarcinoma; WGCNA, Weighted Gene Correlation Network Analysis.

A total of 1971 genes were identified as DEGs after differential expression analysis (Figure 2D). Of these, 902 were up-regulated genes, while 1,069 were down-regulated (Figure 2D). GO analysis revealed that the DEGs were mainly enriched in the biological processes of the humoral immune response, complement activation, and protein activation (Figure 2E). KEGG analysis indicated that the DEGs were mainly enriched in cell adhesion molecules, cell cycle, and complement and coagulation cascades (Figure 2F). Next, we performed WGCNA to identify DEGs involved in LUAD development and progression. In the process of co-expression network construction, we observed that the soft thresholding power β was 5 when the fit index of scale-free topology reached 0.90 (Figure 3A). Nine modules were identified based on the average linkage hierarchical clustering and the soft thresholding power (Figure 3B). We observed that the turquoise module was significantly correlated with LUAD development according to the correlation coefficient and *p*-value (Figure 3C). Ultimately, 329 common genes, which are both marker genes and WGCNA module genes, were selected to construct an expression matrix for further analysis.

### Different Molecular Subtypes Identification

**FIGURE 4:**
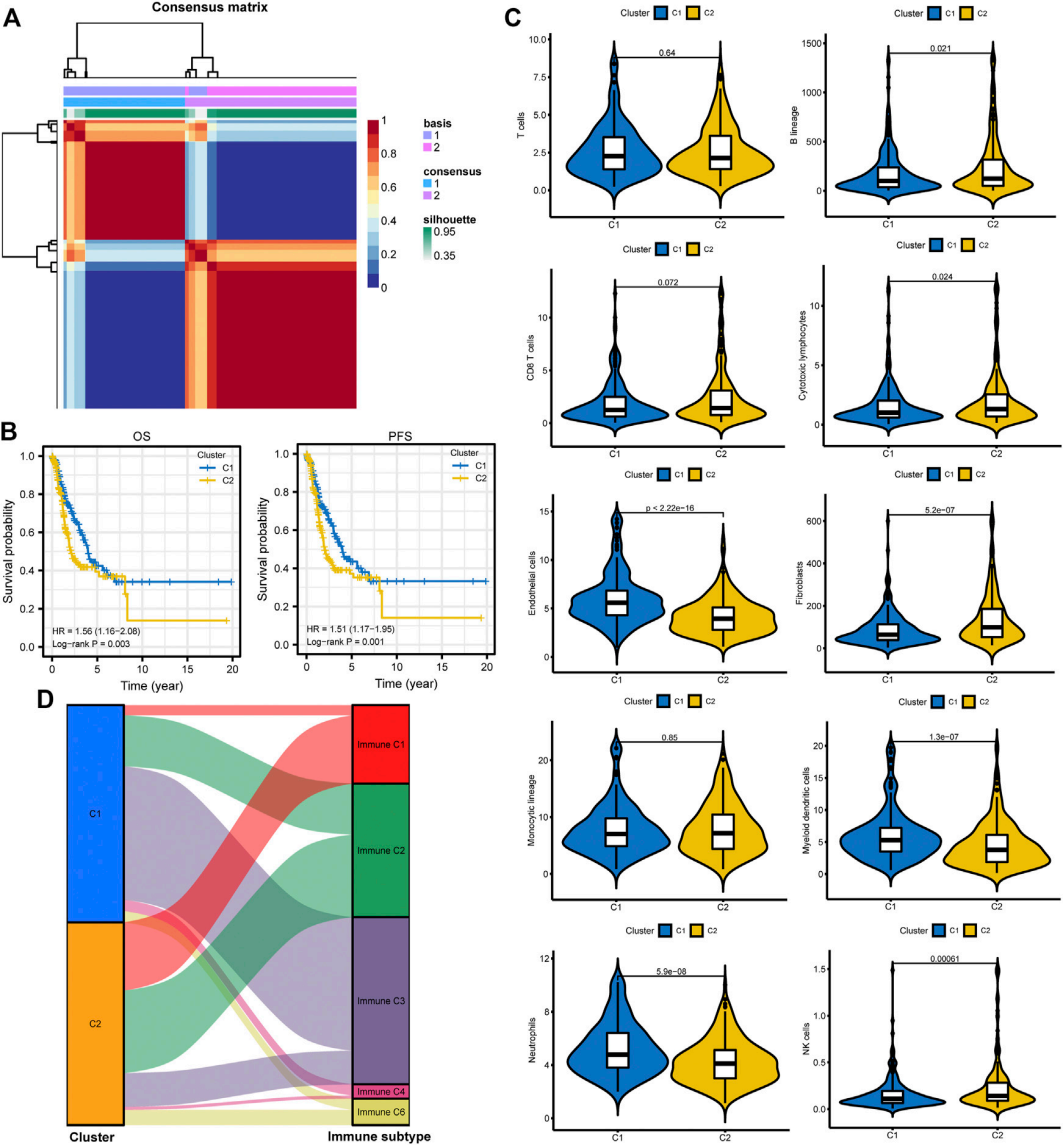
Different subtype identification and clinical relevance analysis. **(A)** Two different subtypes were identified via the NMF algorithm. **(B,C)** The relationship between different subtypes and OS and PFS of LUAD. **(D)** TME composition between different subtypes. **(E)** Sankey plot to show the association between different subtypes and immune subtypes. NMF, non-negative Matrix Factorization; OS, overall survival; PFS, progression-free survival; LUAD, lung adenocarcinoma; TME, tumor microenvironment.

All patients were divided into two clusters according to relevant parameters after NMF (Figure 4A; Supplementary Figure S3). It showed that patients in cluster 2 were correlated with poor OS and PFS than patients in cluster 1 (Figure 4B). The MCPcounter algorithm was used to estimate the infiltration of the immune cells in different clusters. We found that the infiltration levels of endothelial cells, myeloid dendritic cells, and neutrophils were significantly higher in cluster 1 (Figure 4C). However, cluster 2 had higher infiltration levels of B lineage, cytotoxic lymphocytes, fibroblasts, and NK cells (Figure 4C). Besides, the Sankey plot was also applied to investigate the relationship between different immune subtypes and clusters. It showed that patients in cluster 1 are mainly classified into Immune C3 (inflammatory) subtype (Figure 4D). However, patients in cluster 2 are mainly classified into Immune C1 (wound healing), Immune C2 (IFN-gamma dominant), and Immune C6 (TGF-beta dominant) subtypes (Figure 4D).

### Prognostic Model Construction and Validation

**FIGURE 5:**
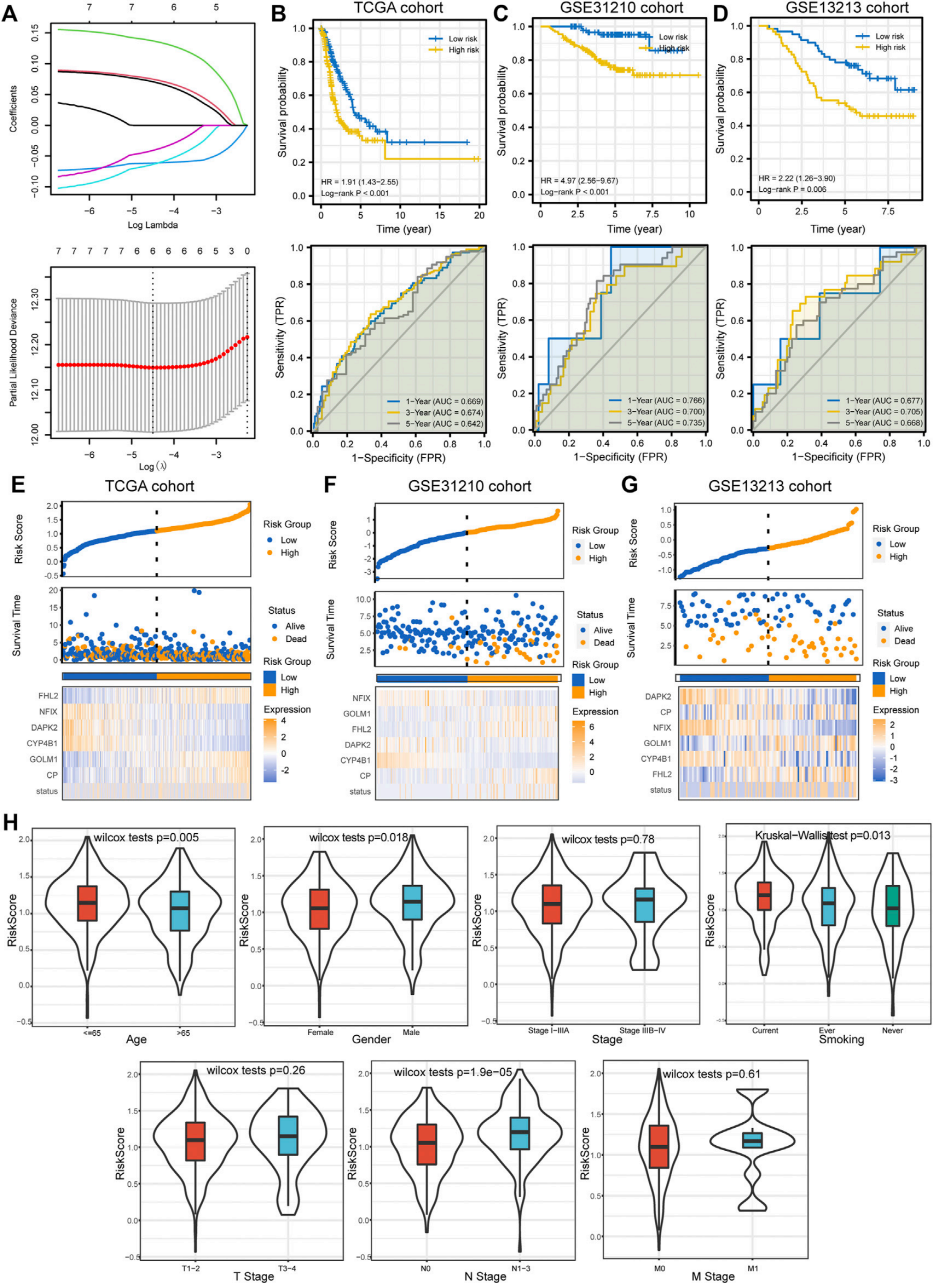
Prognostic model establishment and validation for patients with LUAD. **(A)** Six DEGs were selected for multivariate analysis via LASSO regression analysis. **(B–D)** Survival curves and ROC curves evaluate the risk stratification ability and predictive ability of the constructed risk model in the TCGA**,** GSE31210, and GSE13213 cohorts. **(E–G)** Risk plots to illustrate the survival status of each sample in the TCGA**,** GSE31210, and GSE13213 cohorts. **(H)** The relationship between risk score and common clinicopathological characteristics of LUAD. LUAD, lung adenocarcinoma; DEGs, differentially expressed genes; LASSO, Least Absolute Shrinkage and Selection Operator; ROC, receiver operating characteristic curve; TCGA, The Cancer Genome Atlas.

We performed univariate Cox regression analysis to identify potential prognostic DEGs for LUAD in the TCGA cohort. Seven genes were identified as prognostic DEGs. Then, LASSO regression analysis was performed to reduce the number of DEGs in the final risk model, with six genes were identified through this step (Figure 5A). Ultimately, six genes were recognized as independent prognostic DEGs via multivariate Cox analysis, including *CP*, *GOLM1*, *CYP4B1*, *DAPK2*, *NFIX*, and *FHL2*. According to their coefficients, we calculated the risk score according to the following formula: risk score= expression level of *CP* * 0.088 + expression level of GOLM1* 0.15 + expression level of *CYP4B1* * (−0.064) + expression level of *DAPK2* * (−0.082) + expression level of *NFIX* *(−0.059) + expression level of *FHL2* * 0.086. All patients were divided into high- and low-risk groups according to the median value of risk score. The survival curve showed that patients in the high-risk group were associated with the worse OS when compared with patients in the low-risk group (Figure 5B). Besides, it revealed that the risk score had good performance in predicting the OS in these individuals in the TCGA cohort (AUC for 1-, 3-, and 5 years OS: 0.669, 0.674, and 0.642; Figure 5B). Consistently, we observed similar results in the GSE31210 cohort and GSE13213 cohort (Figures 5C,D). The risk plots were generated to show detailed survival outcomes of each patient in the TCGA cohort and external validation cohorts (Figures 5E–G).

### Clinical Relevance, Enrichment Analysis, and Mutation Landscape Between High- and Low-Risk Groups

**FIGURE 6:**
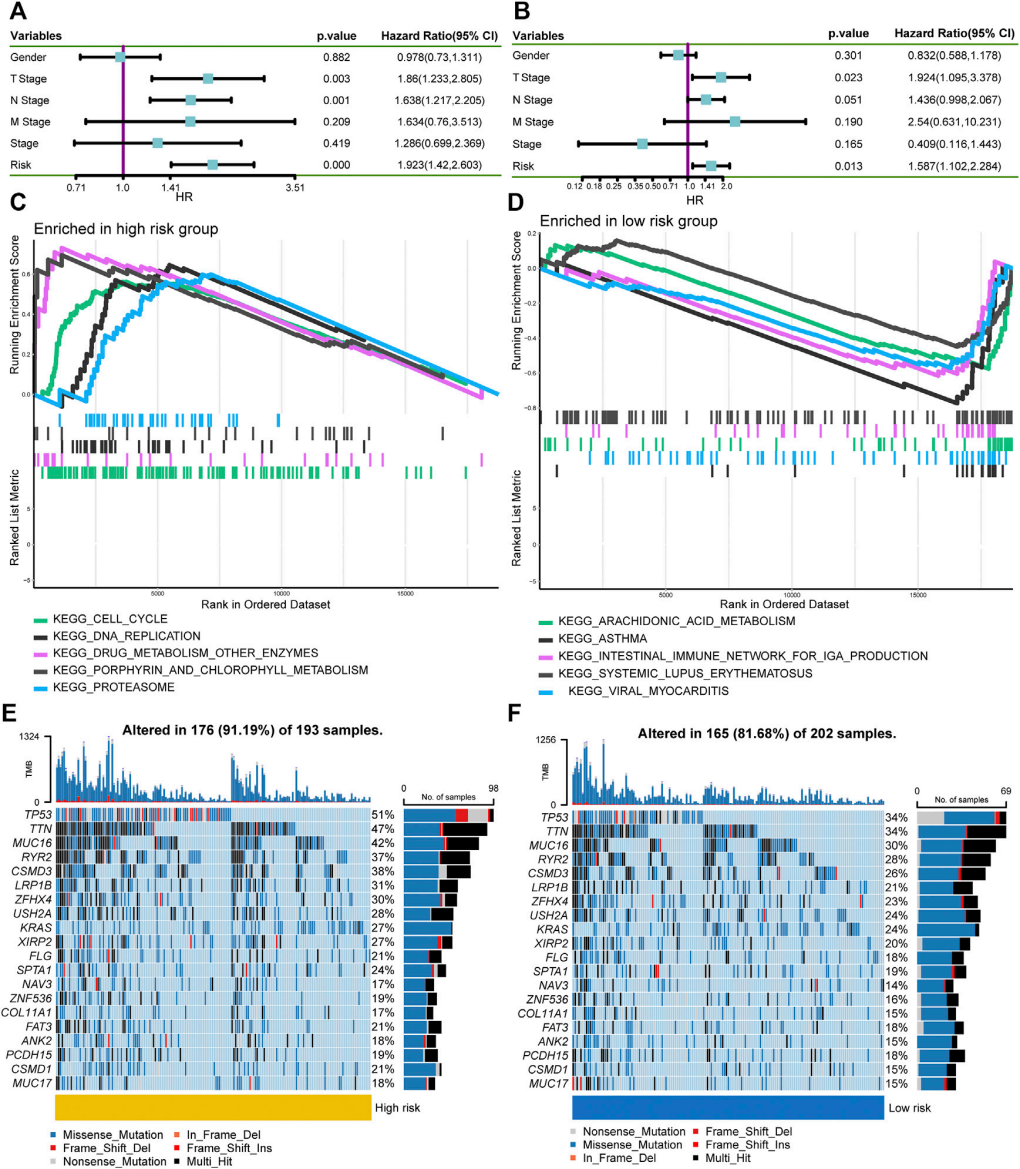
Independent prognostic ability evaluation, pathway enrichment analysis, and gene mutation landscape analysis. **(A,B)** The univariate and multivariate Cox regression analysis demonstrates the risk score’s independent prognostic ability. **(C,D)** GSEA to investigate the biological processes and pathways enriched in high- and low-risk groups. **(E,F)** Waterfall plots summarize the gene mutation landscape in high- and low-risk groups. GSEA, Gene Set Enrichment Analysis.

Next, we investigated the relationship between the risk score and clinicopathological characteristics, suggesting that younger patients, males, current smokers, and positive lymph nodes status were correlated with higher risk scores (Figure 5H). We also performed single factor and multi-factor Cox analyses to determine whether the risk score could be an independent prognostic factor for LUAD patients compared with other common clinicopathological parameters. We observed that the risk score could serve as an independent prognostic factor for these individuals (Figures 6A,B). Furthermore, we performed GSEA analysis to identify the most significantly enriched pathways between the two groups. We found that genes in the high-risk group significantly enriched in cell cycle and DNA replication (Figure 6C). However, genes in the low-risk group significantly enriched in arachidonic acid metabolism (Figure 6D). Afterward, we generated two waterfall plots to explore the detailed gene mutation characteristics between high- and low-risk groups. We identified that *TP53*, *TTN*, and *MUC16* were the most frequently mutated genes in high- and low-risk groups (Figures 6E,F). Besides, we also observed that the high-risk group harbored a more frequent *TP53* mutation rate than the low-risk group (Figures 6E,F).

### The Immune Function Between High- and Low-Risk Groups

**FIGURE 7:**
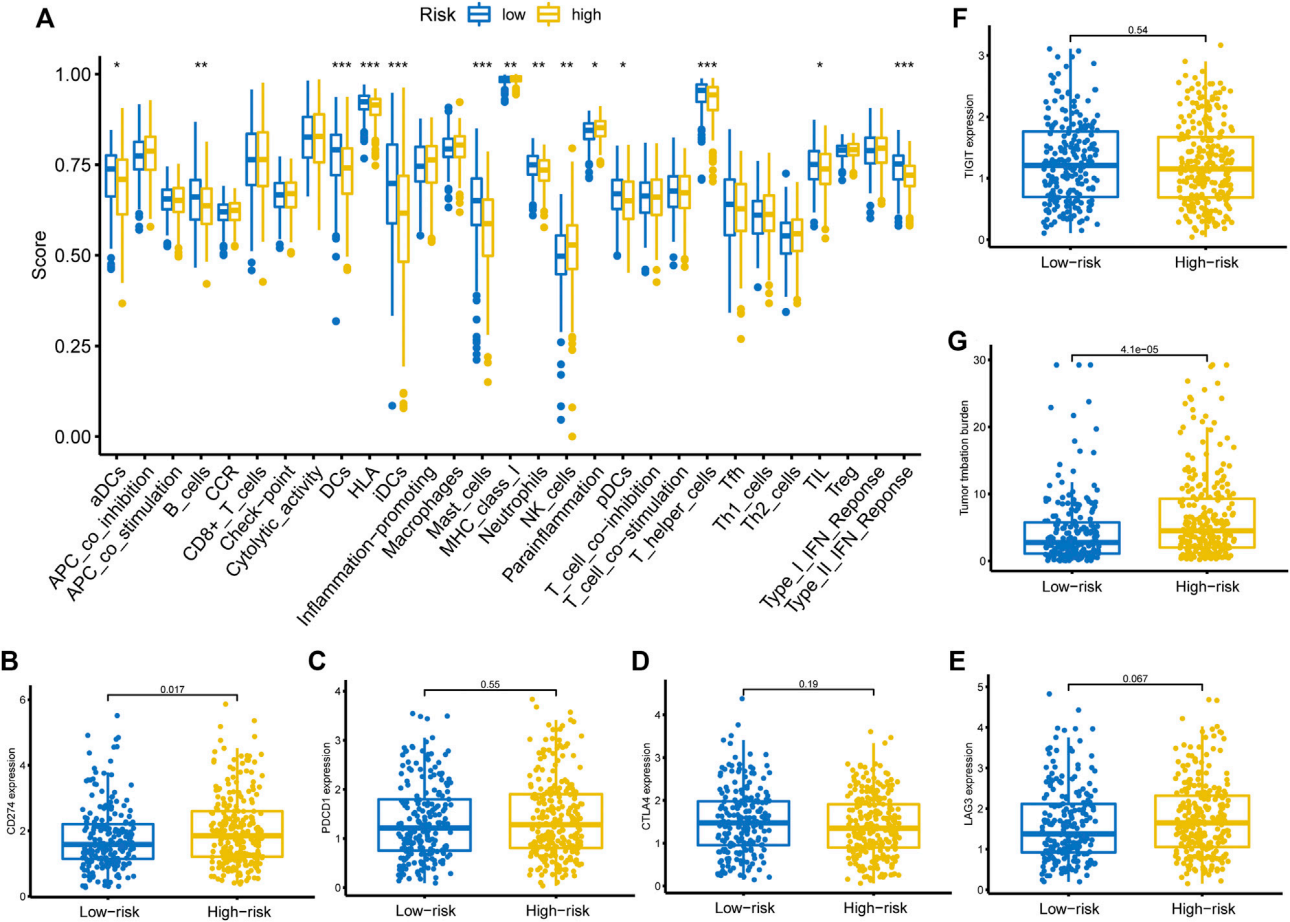
Immune function, ICIs related genes expression pattern, and TMB between different risk groups. **(A)** Immune cells infiltration score and immune-related pathways activity in the low- and high-risk groups estimated by ssGSEA. **(B–F)** The correlation between the risk score and the expression level of *CD274*, *PDCD1*, *CTLA4*, *LAG3*, and *TIGIT*. **(G)** The relationship between the risk score and TMB. ICIs, immune checkpoint inhibitors; TMB, tumor mutation burden, ssGSEA, single-sample gene set enrichment analysis.

We then adopted ssGSEA to estimate the infiltrating score of immune cells and the activity of immune-related pathways in different risk groups. The results demonstrated that the infiltration levels of DCs, B cells, Mast cells, NK cells, T helper cells, and TIL were significantly different in the two groups (Figure 7A). Meanwhile, the two groups also had different scores of MHC class I, parainflammation, and Type II IFN response (Figure 7A). Subsequently, we investigated the correlation between the risk score and the expression level of common ICIs related genes. The results revealed that a higher risk score was significantly associated with up-regulation of *CD274* (*PD-L1*) (Figure 7B). Nevertheless, there was no significant statistical difference between the risk score and *PDCD1* (Figure 7C), *CTLA4* (Figure 7D), *LAG3* (Figure 7E), and *TIGIT* (Figure 7F) expression. Besides, we also observed that a higher risk score was positively correlated with a higher TMB value (Figure 7G).

## Discussion

This study developed a prognostic model for LUAD patients by integrating 10× scRNA-*seq* and bulk RNA-*seq* data. We found that the constructed prognostic model can effectively stratify patients into high- and low-risk groups in the TCGA and GEO cohorts. Furthermore, we also explored the clinical relevance, mutation landscape, and tumor immune microenvironment (TME) in different groups. We noticed that a higher risk score was significantly correlated with a more frequent *TP53* mutation rate, up-regulation of *PD-L1*, and higher TMB value. These results support that patients with higher risk scores could have potential clinical benefits from immunotherapy. Moreover, we identified two distinct subtypes using the NMF algorithm. We observed that different clusters have distinct prognoses and TME components. Cluster 2 was correlated with worse clinical outcomes and high infiltration levels of fibroblasts. Accumulating studies have shown that cancer-associated fibroblasts (CAFs) could transfer lipid to the TME to support cancer cell growth (Lopes-Coelho et al., 2018; Gong et al., 2020; Ma and Zhang, 2021). Recently, Gong et al. elucidated that reprogramming of lipid metabolism in CAFs potentiates migration of colorectal cancer cells through *in vivo* and *in vitro* experiments (Gong et al., 2020). Furthermore, we found that patients in cluster 2 are mainly classified into Immune C1, Immune C2, and Immune C6 subtypes, which are correlated with more aggressive immune infiltrates and worse prognosis (Tamborero et al., 2018; Zhang et al., 2020). On the contrary, patients in cluster 1 are mainly classified into the Immune C3 subtype, associated with a more favorable immune composition and better clinical outcomes (Tamborero et al., 2018; Zhang et al., 2020).

We identified six hub genes to develop the prognostic model through LASSO and Cox regression analyses, including *CP*, *GOLM1*, *CYP4B1*, *DAPK2*, *NFIX*, and *FHL2*. Ceruloplasmin (*CP*) is a multicopper ferroxidase that mainly utilizes the redox activity of copper to oxidize ferrous iron, facilitating iron efflux *via FPN1* (Chen F. et al., 2021). A previous study reported that *CP* is up-regulated in LUAD samples and correlated with poor clinical stage and survival outcome in these patients (Matsuoka et al., 2018). *GOLM1* belongs to the Golgi-associated protein and is a crucial promoter of liver cancer growth and metastasis (Mao et al., 2010). Numerous studies indicated that *GOLM1* is up-regulated in LUAD and can serve as an unfavorable prognostic factor (Liu et al., 2018; Yang et al., 2018; Zhao M. et al., 2021; Song et al., 2021). Song et al. reported that overexpression *GOLM1* enhances lung cancer aggressiveness via inhibiting the formation of *P53* tetramer (Song et al., 2021). Although *GOLM1* has been previously regarded as a diagnostic marker of liver cancer, it is an independent prognostic factor for liver cancer (Mao et al., 2010). In a recent study, Ye et al. revealed that *GOLM1* could drive hepatocellular carcinoma metastasis by modulating EGFR /growth-factor-responsive receptor tyrosine kinase (RTK) cell-surface recycling (Ye et al., 2016). *CYP4B1* is a drug-metabolizing enzyme gene. Several studies detected the mRNA expression level of *CYP4B1* in lung cancer samples and its corresponding paraneoplastic samples (Czerwinski et al., 1994; Tamaki et al., 2011). Tamaki et al. indicated that *CYP4B1* polymorphism is not correlated with lung cancer risk. Therefore, further studies need to be performed to evaluate the expression level of *CYP4B1* in LUAD and its prognostic significance. Death-associated protein kinase (*DAPK*) is the Ser/Thr kinases family member. It has been reported that *DAPK* family proteins play vital roles in mediating apoptosis and function as tumor suppressors in various malignancies (Chen et al., 2014; Jin M. et al., 2021). Interestingly, Jin et al. elucidated that cigarette smoking induces aberrant N6-methyladenosine of *DAPK2* to promote lung cancer progression by activating NF-κB pathway (Jin M. et al., 2021). Nuclear factor IX (*NFIX*) serves as a master regulator, and its expression is associated with 17 genes involved in the migration and invasion pathways, including interleukin-6 receptor subunit β (*IL6ST*), metalloproteinase inhibitor 1 (*TIMP1*), and integrin β-1 (*ITGB1*) (Rahman et al., 2017). In a recent study, Zhao et al. indicated that long non-coding RNA *SNHG3* promotes the development of lung cancer via the miR-1343-3p/*NFIX* pathway (Zhao L. et al., 2021). The four and a half LIM domains 2 (*FHL2*) is a multifunctional scaffolding protein regulating signaling cascades and gene transcription (Wang et al., 2020). Numerous studies have revealed that *FHL2* is an adverse prognostic factor of gynecological malignancies (Wang et al., 2020). However, no study reported the expression level and prognostic significance of *FHL2* in lung cancer.

Subsequently, all patients were divided into low- and high-risk groups by integrating the six hub genes. Two external validation cohorts were also used to verify its predictive ability, with consistent results were observed in these two cohorts. Besides, we identified that the constructed prognostic model has independent predictive ability in predicting the OS of LUAD patients. We then investigated the gene mutation landscape and immune function in different risk groups. We identified that the high-risk group harbored a more frequent *TP53* mutation rate than the low-risk group. Numerous studies identified that *TP53* mutation is closely correlated with treatment resistance and terminal prognosis in lung cancer (Steels et al., 2001; Viktorsson et al., 2005; Xu et al., 2020). However, many studies revealed that *TP53* mutation was significantly correlated with remarkable clinical benefit from PD-1 inhibitors for patients with LUAD since it increases TMB, up-regulates *PD-L1* expression, and remodels TME (Dong et al., 2017; Skoulidis and Heymach, 2019; Xu et al., 2020). Hence, we investigated the relationship between the risk score and TMB value and *PD-L1* expression level. Not surprisingly, it indicated that a higher risk score was significantly correlated with higher TMB value and *PD-L1* expression level. Recently, Yi et al. investigated the regulation of *PD-L1* expression in the TME, suggesting that the expression of *PD-L1* is regulated by numerous factors, including inflammatory stimuli and oncogenic pathways at the levels of transcription, post-transcription, and post-translation (Yi et al., 2021b). Besides, they indicated that a comprehensive framework containing multiple surrogate markers such as TMB would be valuable for selecting patients and predicting outcomes (Yi et al., 2021b). Taken together, patients with higher risk scores could have a potential survival benefit from immune checkpoint blockades treatment. The constructed prognostic model might be a potential predictive biomarker for patients who received immunotherapy. To our knowledge, this is the first study that constructed and validated a prognostic model for LUAD by integrating 10× scRNA-*seq* and bulk RNA-*seq* data. Besides, two external validation cohorts were also used to verify its performance in predicting the OS of these patients. Nevertheless, there are several inevitable shortcomings in our study. First, all these results were obtained from the bioinformatic analysis, and experimental validation needs to be performed in the future. Second, searching for effective prognostic and predictive biomarkers for patients with malignancy is an arduous task for us and needs a long way to go. Our study developed a novel biomarker and provided potential insights in this area. However, well-designed prospective studies are warranted in the future to address this issue.

## Conclusion

This study constructed and validated a prognostic model for LUAD by integrating 10× scRNA-*seq* and bulk RNA-*seq* data. Besides, we identified two distinct subtypes in this population, with different prognosis and immune characteristics being observed in them. The higher risk score was correlated with poor survival outcomes but associated with a more frequent *TP53* mutation rate, higher TMB value, and up-regulation of *PD-L1*. Our prognostic model might be a potential biomarker for LUAD patients’ risk stratification and treatment response prediction. Well-designed prospective studies are warranted in the future to verify our findings.

## Data Availability

The original contributions presented in the study are included in the article/Supplementary Material, further inquiries can be directed to the corresponding authors.
