# Supplementary material for: Integration of Single-Cell RNA Sequencing and Bulk RNA Sequencing Data to Establish and Validate a Prognostic Model for Patients With Lung Adenocarcinoma
Source: Front Genet. 2022 Jan 27;13:833797. doi: 10.3389/fgene.2022.833797 (PMC8829512; doi:10.3389/fgene.2022.833797)
Supplement: Supplementary file 1 [file DataSheet1.PDF]

Table S1. The detailed clinical characteristics of patients in the TCGA and GEO cohorts.

Variables	TCGA cohort	GSE31210 cohort	GSE13213 cohort
Age			
⩾65	274	64	41
<65	217	177	76
NA	19		
Gender			
Female	271	130	57
Male	239	116	60
Clinical stage			
I-IIIA	470	226	112
IIIB-IV	33	NA	5
NA	7	20	-
T stage		NA	
T1-2	444		104
T3-4	66		13
N stage		NA	
N0	335		87
N1-3	175		30
M stage		NA	
M0	349		117
M1	22		0
NA	139		-
Smoking history			NA
Current	122	NA	
Ever	304	123	
Never	70	123	
NA	14	-	
Survival status			
Death	180	35	29
Alive	321	191	58
NA	9	20	0

Figure S1

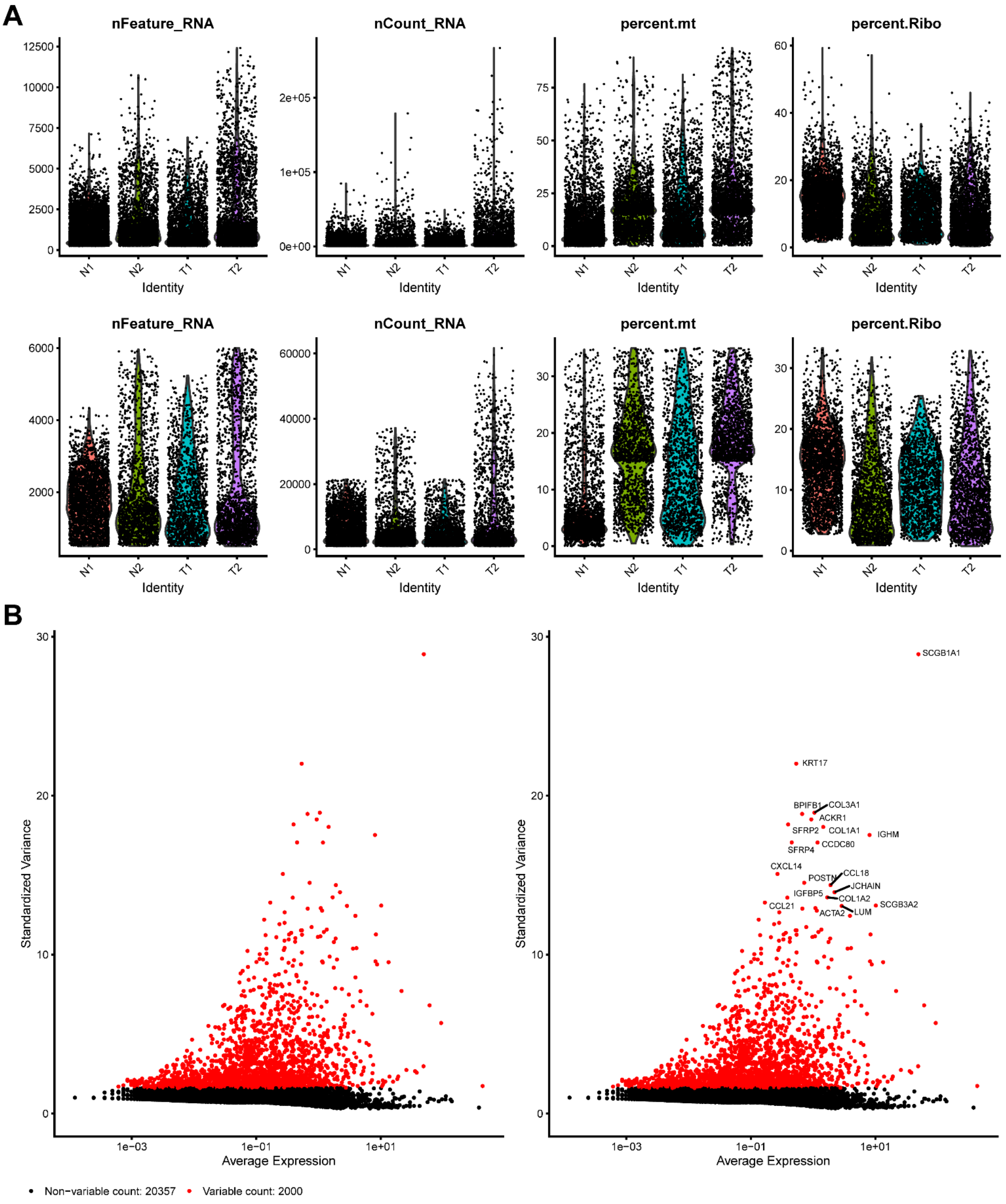
A
nFeature_RNA
nCount_RNA
percent.mt
percent.Ribo
Identity
N1
N2
T1
T2
B
Standardized Variance
Average Expression
SCGB1A1
KRT17
BPIFB1
COL3A1
ACKR1
SFRP2
COL1A1
IGHM
CCDC80
SFRP4
CXCL14
POSTN
CCL18
JCHAIN
IGFBP5
COL1A2
CCL21
SCGB3A2
ACTA2
LUM
Non−variable count: 20357
Variable count: 2000

Figure S2

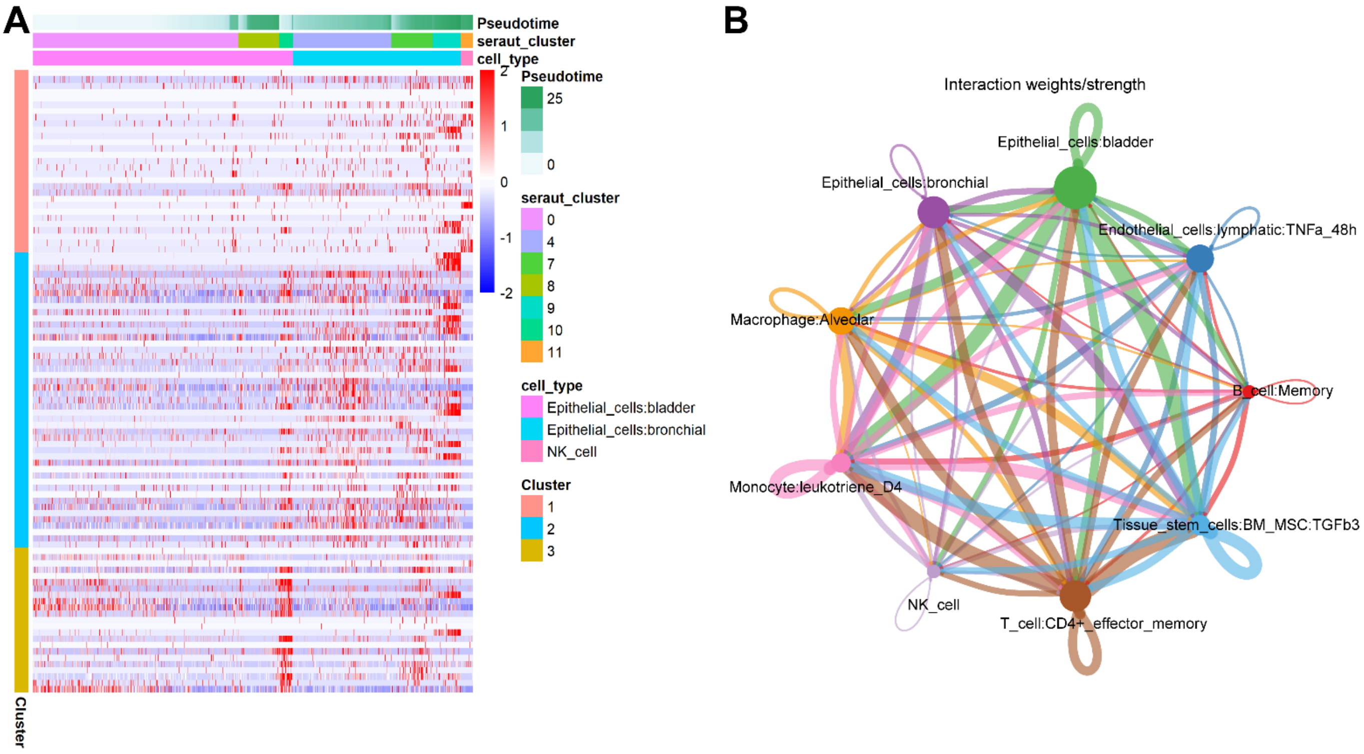
A
Pseudotime
seraut_cluster
cell_type
2
1
0
-1
-2
Pseudotime
25
0
seraut_cluster
0
4
7
8
9
10
11
cell_type
Epithelial_cells:bladder
Epithelial_cells:bronchial
NK_cell
Cluster
1
2
3
Cluster
B
Interaction weights/strength
Epithelial_cells:bladder
Epithelial_cells:bronchial
Endothelial_cells:lymphatic:TNFa_48h
Macrophage:Alveolar
B_cell:Memory
Monocyte:leukotriene_D4
Tissue_stem_cells:BM_MSC:TGFb3
NK_cell
T_cell:CD4+_effector_memory

Figure S3

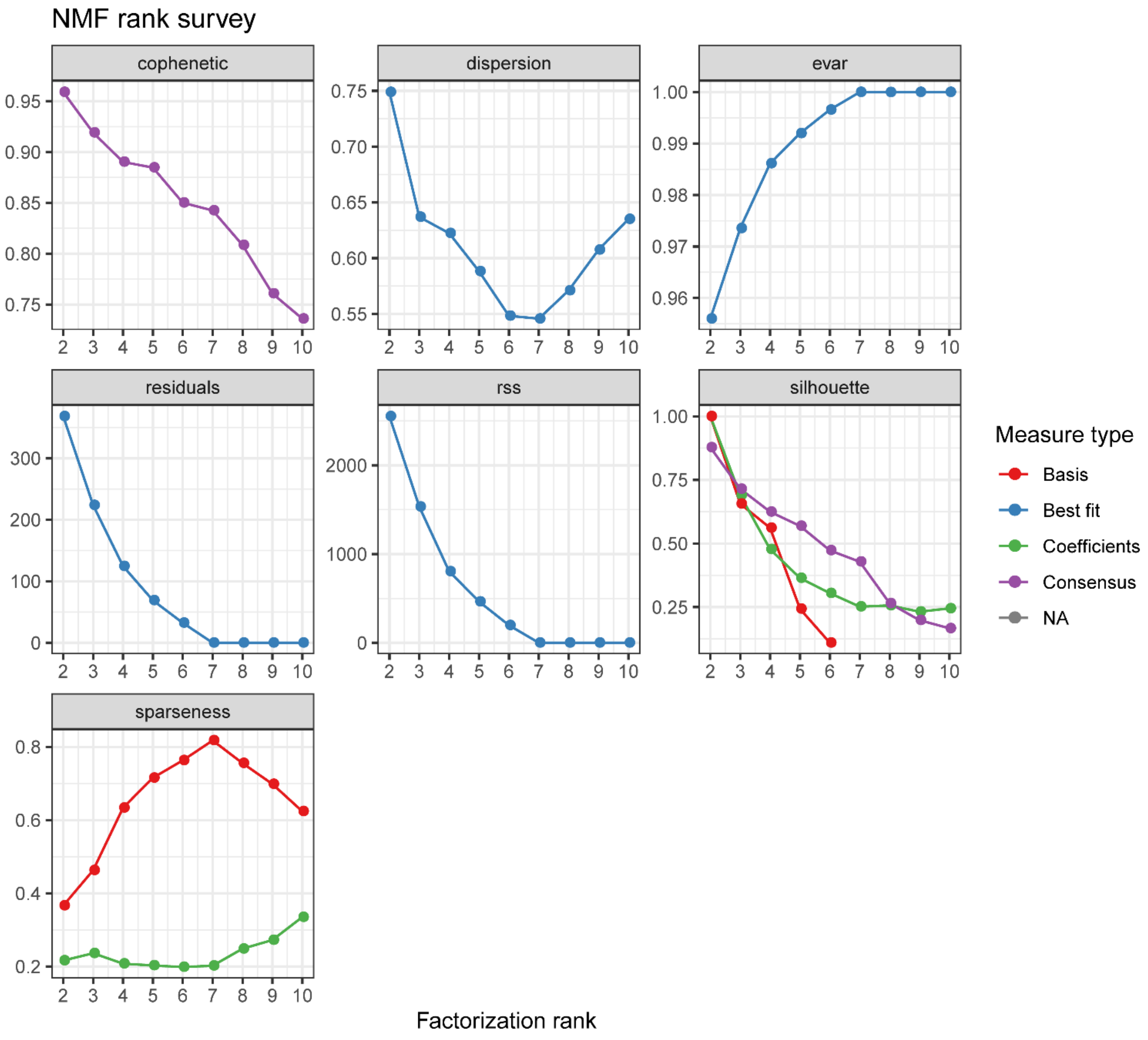

NMF rank survey
cophenetic
dispersion
evar
residuals
rss
silhouette
sparseness
0.95
0.90
0.85
0.80
0.75
0.70
0.65
0.60
0.55
1.00
0.99
0.98
0.97
0.96
300
200
100
0
2000
1000
0.50
0.25
0.8
0.6
0.4
0.2
2 3 4 5 6 7 8 9 10
Measure type
Basis
Best fit
Coefficients
Consensus
NA
Factorization rank
